# Nature’s pre-installed helpers: diverse seed endophytes enhance rice nitrogen use efficiency

**DOI:** 10.3389/fpls.2025.1709648

**Published:** 2026-01-20

**Authors:** Ruimin Lao, Shaoxing Fang, Wenjun Fang, Zhiwei Zhao, Haiyan Li, Tao Li

**Affiliations:** 1 Medical School, Kunming University of Science and Technology, Kunming, China; 2 State Key Laboratory for Conservation and Utilization of Bio-Resources in Yunnan, Yunnan University, Kunming, China

**Keywords:** rice seed endophytes, microbial communities, rata taxa, core taxa, plant growth promotion (PGP), nitrogen use efficiency (NUE)

## Abstract

Nitrogen is a key limiting factor for crop growth, and improving nitrogen use efficiency (NUE) is critical for achieving high crop yields. In this study, both culture-independent and culture-dependent approaches were employed to systematically analyze the community composition and functional traits of seed endophytic bacteria in rice varieties with contrasting NUE. The results revealed diverse endophytic bacterial communities across the four rice varieties, with Shannon indices ranging from 2.95 to 3.23. However, significant compositional differences were observed among varieties. Rare taxa accounted for over 51% of operational taxonomic units (OTUs) in each variety and were the primary drivers of community diversity and differentiation. In contrast, core taxa (shared OTUs) were highly conserved across varieties, largely composed of abundant taxa (OTUs > 39%, total relative abundance > 93%), and occupied central positions in co-occurrence networks, thereby contributing to community stability. Five representative strains exhibited diverse plant growth-promoting (PGP) traits *in vitro*, including siderophore production, phosphate solubilization, and indole - 3 - acetic acid (IAA) synthesis. These functions were partially redundant, but individual strains exhibited distinct strengths, indicating functional complementarity. Inoculation experiments demonstrated that all strains improved rice growth, nitrogen accumulation, and NUE, with their effectiveness modulated by both strain identity and nitrogen availability. This study reveals rice seed endophytic bacteria as “natural microbial allies” that support host growth and adaptation under low-nitrogen conditions. These endophytes represent valuable microbial resources for the development of next-generation biofertilizers in sustainable agriculture.

## 1 Introduction

Nitrogen, as an essential macronutrient for plant growth and development, plays a critical role in determining crop performance and yield. In agricultural ecosystems, nitrogen use efficiency (NUE) varies significantly both between and within crop species (Fan et al., 2020). For instance, at the subspecies level, indica and japonica rice exhibit marked divergence in the regulatory pathways of nitrate uptake and assimilation, such as the transcription factor OsWRKY23 (Zhang et al., 2025a). Even at the variety level, the maize variety “Denghai605” shows significantly higher NUE than “Ludan981” under both nitrogen-rich and nitrogen-deficient conditions (Shao et al., 2022). Consequently, integrated strategies that combine traditional phenotypic selection, molecular breeding, and artificial intelligence-based prediction have become effective approaches for developing high-NUE crop varieties and mitigating nitrogen fertilizer pollution (Anas et al., 2020; Zhang et al., 2025b). Notably, plant-associated microbiomes, often referred to as the plant’s “second genome,” have demonstrated great potential in enhancing crop NUE. Harnessing the functional capabilities of these microbiomes has emerged as a promising frontier in breeding high-NUE varieties of economically important crops (Xu et al., 2025).

Plant-associated microbiomes, especially functional microbes in the rhizosphere, have been shown to improve host NUE through diverse interaction mechanisms. On the one hand, both free-living and symbiotic nitrogen-fixing bacteria contribute directly to nitrogen availability via biological nitrogen fixation (Guo et al., 2023). On the other hand, plant-associated microbes can also modulate the expression of host genes involved in nitrogen uptake and assimilation, thereby enhancing nitrogen metabolism. For instance, the arbuscular mycorrhizal (AM) fungus Rhizophagus irregularis promotes the uptake and assimilation of ammonium (NH_4_^+^) in Medicago sativa by upregulating key mycorrhizal nitrogen transporter genes, RicPSI and RicARI (Wang et al., 2024). Similarly, the plant growth-promoting bacterium Azospirillum brasilense can significantly enhance nitrogen assimilation efficiency in rice (Oryza sativa cv. Nipponbare) by upregulating the expression of multiple genes, including nitrate transporter and nodulin gene MtN3 (Thomas et al., 2019). Moreover, rhizosphere microbial colonization can influence root system architecture, thereby indirectly facilitating nitrogen uptake. For example, Wu et al. (2025) found that Pseudomonas spp. stimulate lateral root development in Leuce poplar through the secretion of indole - 3 - acetic acid (IAA), expanding root surface area and enhancing nitrogen acquisition. In addition, high-NUE varieties have the capacity to selectively enrich specific functional microbial taxa in the rhizosphere, thereby reshaping microbial community composition and accelerating nitrogen transformation rates in soil, ultimately improving plant NUE. ^15^N isotope tracing studies have shown that high-NUE rice varieties can restructure their rhizosphere microbiomes compared to bulk soil, leading to faster nitrogen turnover and improved nitrogen use efficiency (Chen et al., 2025). Collectively, these findings provide important theoretical foundations for microbial strategies to improve plant NUE.

Although rhizosphere microbial communities play a significant role in enhancing crop NUE, their functional performance is often constrained by multiple factors, including environmental conditions (e.g., soil moisture and nutrient availability) and host plant genotypes (Rodriguez et al., 2009). In particular, the colonization compatibility between microbes and host tissues is critical for the expression of microbial functions. For example, Wu and Chang (2024) demonstrated that the differential colonization ability of the crown rot–antagonistic strain Bacillus altitudinis in various soybean varieties significantly affected the level of disease resistance. In contrast, seed-associated microbes—an integral part of the plant microbiome—possess distinctive and inherently stable advantages. First, the vertical transmission of seed endophytes ensures the stable fidelity of the microbiome during intergenerational transfer (Johnston-Monje et al., 2016). Second, as a primary inoculum, the seed microbiome can colonize the root system early during seedling establishment, preemptively occupying ecological niches and forming dominant rhizosphere communities (Shade et al., 2017). Third, seed-borne microbes serve as natural microbial allies, playing key roles in host development and in mediating adaptive responses to environmental stresses (Truyens et al., 2015).

As a globally important crop with a high dependency on nitrogen, rice presents significant research value for exploring the composition of its seed microbiome and its functional role in enhancing host NUE. In this study, we conducted a comparative analysis of high- and low- NUE rice varieties and proposed the following hypotheses: (1) seeds act as reservoirs of diverse endophytic bacteria, and the seed microbiome composition varies across rice varieties; (2) seed-borne endophytes possess functional traits that contribute to improving host NUE; and (3) the functional characteristics of seed endophytes are consistent with, and may extend, the physiological traits of their host plants. Therefore, investigating the diversity and functional roles of seed-borne ‘primary inoculum’ in rice varieties with contrasting NUE can uncover microbial candidates for improving host NUE and offer new insights into the biological strategies for enhancing nitrogen use in rice.

## 2 Materials and methods

### 2.1 Rice cultivars and nitrogen use efficiency characteristics

In this study, a systematic review of 20 publications from 2010 to 2022 was conducted to identify four widely cultivated rice varieties in China that exhibit significant differences in NUE as candidate materials. These cultivars were grown under greenhouse conditions for 30 days with either normal nitrogen (42.23 mg kg^−1^, N42) or low nitrogen (7.39 mg kg^−1^, N7) treatments. Based on key physiological traits—including biomass, nitrogen accumulation, and NUE—two high-NUE varieties, Tianyouhuazhan (H_TY_) (Feng et al., 2014; Hu et al., 2019) and Y Liangyou 1# (H_Y1_) (Cui et al., 2010); and two low-NUE varieties, Xiushui 134# (L_XS_) (Feng et al., 2014) and Fuyuan 4# (L_FY_) (An et al., 2014) (**Supplementary Table S1**). For downstream analyses, 10 g of seeds from each of the four representative varieties were surface-sterilized by sequential immersion in 75% ethanol for 1 minute, followed by 5 minutes in 10% sodium hypochlorite, and then rinsed 3–5 times with sterile distilled water, according to the protocol of Zhang et al. (2021). The sterilized seeds were then transferred into 5 mL EP tubes and homogenized using a tissue grinder (Jingxin, Shanghai, China) at 50 Hz for 200 s to prepare seed suspensions for further experiments.

### 2.2 DNA extraction and sequencing of seed endophytic bacteria

Total environmental DNA for bacterial community analysis was extracted from the 2 g seed suspensions prepared as described above, using the MolPure^®^ Plant DNA kit (Guangzhou Feiyang Bioengineering Co., Ltd., China), according to the manufacturer’s instructions. DNA was eluted in 50 μl of purified water and quantified using a Nanodrop spectrophotometer (ThermoFisher, Massachusetts, USA). The V5-V6 region of the bacterial 16S rRNA gene was amplified using universal primers 799F (5′-AAC MGG ATT AGA TAC CCK G-3′) and V6R (3′-GGG TTG CGC TCG TTG CG-5′) (Yang et al., 2024). PCR reactions were performed in 20 µL volumes containing 4 µL 5× FastPfu Buffer, 2 µL 2.5 mM dNTPs, 0.8 µL of each primer (5 µM), 0.4 µL FastPfu DNA polymerase (Beijing TransGen Biotech Co., Ltd., China), 0.2 µL BSA, and 10 ng of template DNA. The thermal cycling protocol consisted of 3 min at 95°C for pre-denaturation, followed by 30 cycles of 95°C for 30 s, 58 °C for 30 s, and 72°C for 45 s, with a final extension at 72°C for 10 min. PCR products were purified using a PCR Clean-Up Kit (Axygen Co. Ltd., USA) and quantified with a Qubit 4.0 Fluorometer (Thermo Fisher Scientific, USA). Library construction was performed using the NEXTFLEX Rapid DNA-Seq Kit (Shanghai Xinrui Biotechnology Co., Ltd., China), and sequencing was carried out on the Illumina Nextseq2000 platform.

### 2.3 Diversity analysis of seed endophytic bacterial communities

Raw sequencing reads were subjected to quality filtering using Fastp (v0.19.6), followed by paired-end read merging using FLASH (v1.2.11). Sequences with > 97% similarity were clustered into operational taxonomic units (OTUs) using UPARSE (v7.1) (Edgar, 2013). OTU classification and abundance were annotated using the RDP Classifier (v2.11) with a confidence threshold of 97% (Wang et al., 2007). OTUs were categorized as abundant taxa (AT, abundance > 0.1%), intermediate taxa (IT, 0.01% ≤ abundance ≤ 0.1%), and rare taxa (RT, abundance< 0.01%) according to Zhao et al. (2022). Alpha diversity indices were calculated using Mothur (v1.30.2) (Schloss et al., 2009), and Bray-Curtis principal component analysis (PCA) was used to assess community structure similarity (Siebyła et al., 2024). The top 50 most abundant bacterial OTUs were selected for microbial co-occurrence network analysis, and network topological parameters such as modularity and clustering coefficient were calculated using Gephi (version 0.10.1) (Barberán et al., 2012). Bacterial functional prediction was performed with the FAPROTAX database (Louca et al., 2016). Indicator species analysis was conducted on the Majorbio Cloud Bioinformatics Platform (https://www.majorbio.com), and OTUs with IndVal > 0.7 and p < 0.05 were considered cultivar-specific indicators. Based on Yuan et al. (2021), network robustness was assessed using the Top 50 most abundant OTUs per cultivar in R (v4.4.3). Robustness was defined as the proportion of remaining nodes after randomly removing 50% of network nodes. The raw sequence data are deposited in NCBI SRA under BioProject PRJNA1303560.

### 2.4 Isolation and identification of seed endophytes from high NUE varieties

For bacterial isolation, 2 g of seeds from the H_TY_ and H_Y1_ cultivars were surface-sterilized, homogenized, and serially diluted following the procedure described above. Aliquots (100 µL) from 5^−4^ to 5^−4−6^ dilutions were spread onto Luria Bertani (LB), Nutrient Agar (NA), and Reasoner’s 2A (R_2_A) agar plates, and incubated at 37°C overnight, respectively (Ahn et al., 2014; Ezraty et al., 2014; Zhang et al., 2022). Single colonies were selected and identified using 16S rRNA gene amplification with primers 27F (5′-AGA GTT TGA TCC TGG CTC AG-3′) and 1492R (5′-TAC GGC TAC CTT GTT ACG ACT T-3′), and M5 Hiper Mix DNA polymerase (Xi’an Zhuangzhi Biotechnology Co., Ltd., China) (Hou et al., 2018). PCR reactions (20 µL) contained 2 µL DNA template, 0.5 µL primers, and 10 µL enzyme mix. Cycling conditions were: 95°C for 3 min; 35 cycles of 94°C for 25 s, 55°C for 25 s, 72°C for 90 s; final extension at 72°C for 5 min. Sequences were trimmed and clustered at ≥ 99% similarity using SeqMan and Mothur. Representative sequences were taxonomically identified via BLAST searches against the NCBI database. Closely related reference sequences were retrieved from GenBank and aligned with the target sequences in MEGA (v11.0.13). A Neighbor-Joining phylogenetic tree was subsequently constructed, and node reliability was assessed with 1,000 bootstrap replicates (Koester et al., 2019; Tamura et al., 2013).

### 2.5 Plant growth-promoting traits of representative seed endophytic strains

**(1) Phosphate solubilization:** Each strain (OD_600_ = 0.3) was spot-inoculated (2 µL) onto solid National Botanical Research Institute Phosphate (NBRIP) medium supplemented with 5 g/L of either phytate calcium or tricalcium phosphate as the only phosphorus source. Plates were incubated at 37°C for 2 days. The phosphate solubilization ability was assessed by the formation of clear halos around colonies using the cross-line method, in which two perpendicular lines were drawn through the center of the colony and the distance from the colony edge to the halo edge along each line was recorded. For quantitative analysis, 1 mL of each strain was inoculated into 100 mL sterile NBRIP liquid medium and incubated at 37°C, 180 rpm for 3 days with five replicates. A sterile, uninoculated medium served as the control. After centrifugation at 10,000 rpm for 3 min, the pH of the supernatant was measured using a pH meter (FiveEasy Plus, FE28, Shanghai, China). Soluble phosphorus concentrations were determined using the molybdenum blue colorimetric method, following the Chinese national standard NY/T 2017-2011 (Watanabe and Olsen, 1965). OD_700_ was measured with a microplate reader (Molecular Devices, FlexStation 3, Shanghai, China).

**(2) Siderophore production:** Each bacterial strain (OD_600_ = 0.3) was spot-inoculated (2 µL) onto chrome azurol S (CAS)-based double-layer agar plates prepared with LB medium and incubated at 37°C for 48 hours. The presence of orange halos around colonies indicated siderophore production (Pérez-Miranda et al., 2007). For quantitative assessment, 1 mL of bacterial culture was added to 100 mL sterile iron-limited Modified King’s B (MKB) liquid medium and incubated at 37 °C, 180 rpm for 3 days (5 replicates). After centrifugation at 4,500 rpm for 5 min, 1 mL of supernatant was mixed with 1 mL CAS assay solution and incubated at room temperature for 1 hour. Distilled water was used as a blank control. OD_630_ was measured for each sample, and siderophore units (SU) were calculated as follows (Schwyn and Neilands, 1987):


$$\text{SU}\;\left(\%\right)=\frac{100\times \left(Ar-As\right)}{Ar}$$

Where *As* is the OD_630_ value of the treatment group, and *A*r is the OD_630_ value of the control group. Siderophore production capacity was standardized by dividing SU of the bacterial culture.

**(3) Quantification of IAA Production:** IAA production was quantified according to the method described by Carvajal et al. (2024), with minor modifications. 1 mL of bacterial suspension was inoculated into 50 mL of sterilized modified LB liquid medium supplemented with 100 mg·L^−1^ L-tryptophan. Cultures were incubated at 37 °C with shaking at 180 rpm for 3 days. Each treatment was conducted with five replicates, and an uninoculated medium served as the control. After incubation, cultures were centrifuged at 8000 rpm for 10 min. Then, 50 μL of the supernatant was mixed with an equal volume of Salkowski reagent. In parallel, IAA standards were prepared at concentrations of 0, 10, 20, 30, 40, and 50 mg mL^−1^, each with three replicates, and processed identically. All samples were incubated in the dark at room temperature for 30 min. Absorbance was measured at 530 nm for each sample. A linear regression of standard concentration (x) against absorbance (y) was used to generate the calibration equation (R² = 0.9953). The IAA concentration of each sample was subsequently calculated from the standard curve. Double-distilled water (ddH_2_O) served as a blank control.

### 2.6 Effects of seed endophyte on rice growth and NUE

Well-filled seeds of the low-NUE rice variety L_XS_ (with husks removed) were selected, soaked in sterile water for 8 hours, and then surface-sterilized following the procedure described above. The disinfected seeds were sown onto 1/2 Murashige Skoog (MS) medium supplemented with 1% Plant Preservative Mixture (PPM™, Beijing Coolibor Technology Co., Ltd., Beijing, China) and 0.25% agar. After 2 days of incubation in darkness, the germinated seeds were transferred to a light chamber and cultivated for 2 weeks. Embryo-derived sterile seedlings were obtained by carefully excising the endosperm and removing any adhering MS medium. Uniformly grown sterile seedlings were used as hosts for inoculation with each of five representative endophytic bacterial strains. Autoclaved bacterial inocula (121°C, 20 min) served as negative controls. For the inoculation treatments, a root-dipping method was employed: sterile rice seedling roots were immersed in 10 mL of a bacterial suspension (OD_600_ = 0.3) for pre-inoculation. The pre-inoculated seedlings were then transplanted into pots (9 × 23.5 × 5 cm; top diameter × height × bottom diameter) containing 350 g of growth substrate, after which the remaining bacterial suspension was applied and watered around the root zone to promote effective colonization (Wang and Gottwald, 2017). The growth substrate was composed of commercial vermiculite, farmland soil, and commercial humus soil mixed at a ratio of 50:1:1 (w/w) (organic matter = 12.93 g kg^−1^, total N = 0.216 mg kg^−1^, total P = 4.182 mg kg^−1^, total K = 34.25 mg kg^−1^, available N = 0.0481 mg kg^−1^, available P = 7.544 mg kg^−1^, available K = 75.41 mg kg^−1^, and pH = 6.89). The substrate was sterilized at 121 °C for 120 minutes, with 3 consecutive treatments (24 hours apart each time). Two nitrogen treatments were established by supplementing the substrate with nutrient solution containing urea (Tianjin Fengchuan Chemical Reagent Co., Ltd., Tianjin, China) as the N source: low N (N7, 7.39 mg kg^−1^) and normal N (N42, 42.23 mg kg^−1^). Each pot contained one seedling, with five replicates per treatment. In total, the experiment comprised 2 nitrogen levels × 6 inoculants (5 bacteria + ck) × 6 replicates = 72 pots. Plants were grown for 60 days in a greenhouse under natural light at 15–30 °C and irrigated with 60 mL sterile water every two days. Prior to harvest, plant height, tiller number, fresh leaf number, and number of senescent leaves were recorded. Leaf chlorophyll content was measured using a SPAD-502 Plus chlorophyll meter (Konica Minolta, Japan). After 60 days, both shoot and root tissues were separately harvested for further analysis.

### 2.7 Sample digestion and quantification of nitrogen-related parameters

Following the Chinese national standard “Determination of nitrogen, phosphorus and potassium in plants” (NY/T 2419-2013), plant tissues were digested using the concentrated sulfuric acid–hydrogen peroxide method. Total nitrogen content was then determined using a Kjeldahl nitrogen analyzer (SKD-800, Shanghai Peiou Analytical Instrument Co., Ltd., Shanghai, China), according to the manufacturer’s instructions.

Nitrogen Transport efficiency (NTE), Nitrogen Uptake efficiency (NUpE), Nitrogen Utilization efficiency (NUtE) and Nitrogen Use efficiency (NUE) was calculated using the following formula (Cheng et al., 2011; Liu et al., 2025):


$$\text{Nitrogen Transport Efficiency }\left(\text{NTE},\text{ \%}\right)=\frac{\text{Total shoot N accumulation}}{\text{total plant N accumulation}}\times 100\%$$

$$\text{Nitrogen Uptake Efficiency }\left(\text{NUpE},\text{ \%}\right)=\frac{\text{total plant N accumulation}}{\text{total N investment}}\times 100\%$$

$$\text{Nitrogen Utilization Efficiency }\left(\text{NUtE},\text{ g}/\text{g}\right)=\frac{\text{plant dry matter}}{\text{total plant N accumulation}}$$

Nitrogen Use Efficiency (NUE, g/g)=NUpE × NUtE

### 2.8 Statistical analysis

Statistical analyses were conducted using SPSS software (version 24.0; IBM, Armonk, NY, USA), and data visualization was performed with Origin 2018 (OriginLab, Northampton, MA, USA). Two-way ANOVA was used to evaluate the main and interaction effects of endophyte inoculation and nitrogen level, assuming homogeneity of variance and normality. When significant effects were detected, one-way ANOVA, followed by post-hoc Tukey’s HSD test, was used to detect significant difference in rice biomass, plant height, chlorophyll content, nitrogen concentration, and NUE among the bacterial inoculation treatments and the control (CK) under the same nitrogen level (p < 0.05). Independent-samples t-tests were applied to compare the effects of nitrogen treatments on these parameters in the CK group. Data are expressed as means ± standard error (SE).

## 3 Results

### 3.1 Diversity of seed endophytic bacteria between High- and Low-NUE rice cultivars

#### 3.1.1 Characteristics of seed endophytic bacterial communities

**Figure 1:**
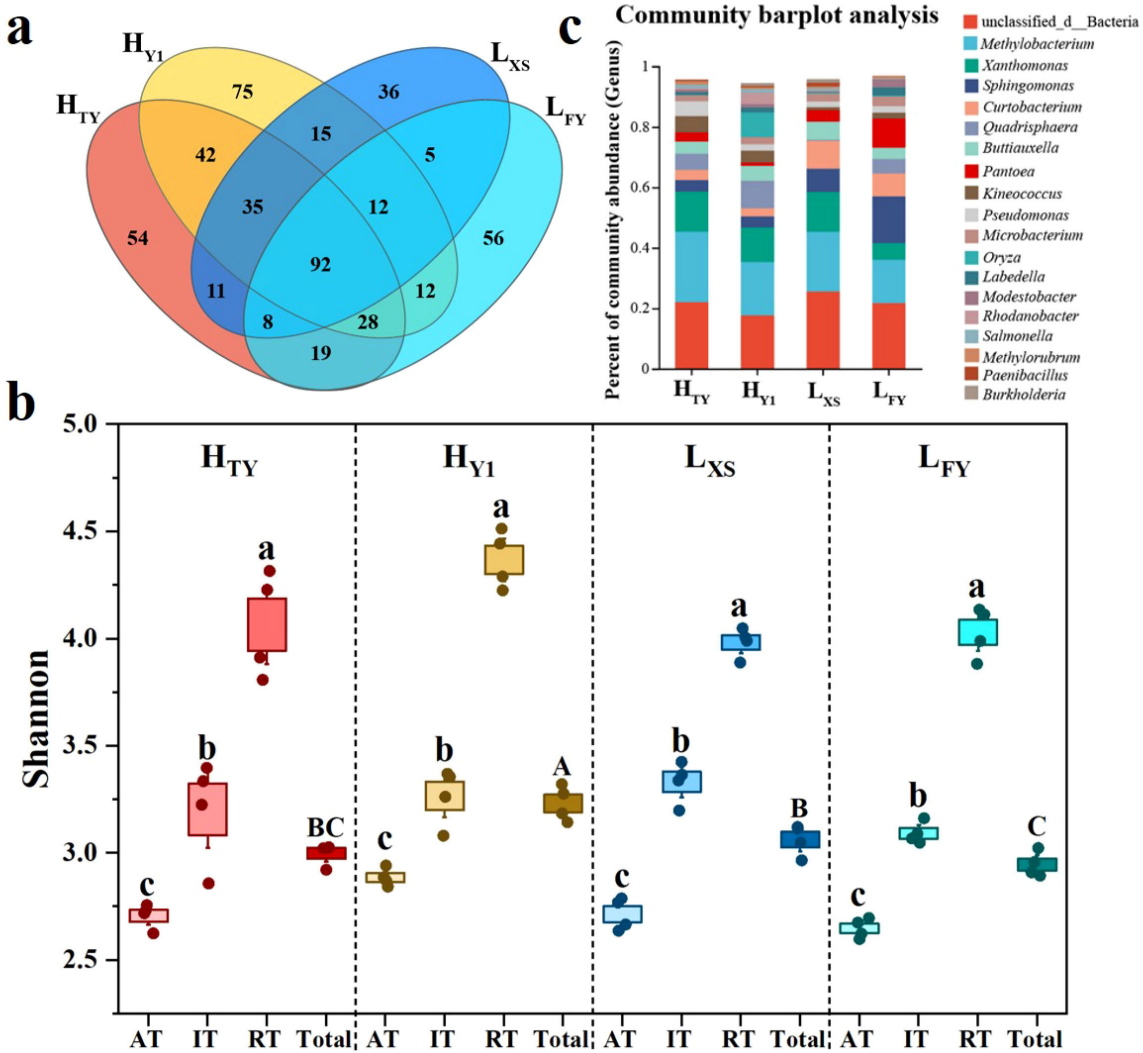
Diversity of seed endophytic bacterial communities in rice cultivars with high (H_TY_, H_Y1_) and low (L_FY_, L_XS_) nitrogen use efficiency. **(a)** Venn diagram, **(b)** Shannon indices, and **(c)** Community composition. AT, IT and RT represent abundant, intermediate and rare bacterial taxa, respectively, while ‘Total’ refers to all taxa in the seed endophytic bacterial communities. Different letters indicate significant differences between groups (*p* < 0.05, one-way ANOVA, *n* = 4).

A total of 500 OTUs of seed endophytic bacteria were annotated across the four rice cultivars, with individual cultivars containing 214 (L_XS_) to 311 (H_Y1_) OTUs. Among these, 92 OTUs (18.4%) were shared across all cultivars, yet accounting for over 94.5% of total abundance, reaching up to 98.4% in L_FY_ variety. Rare taxa (RT) dominated OTU richness, representing over 51% of OTUs in all cultivars, although their total abundance was below 0.6%. In contrast, abundant taxa (AT) comprised only 15.1% (H_Y1_, 47 OTUs) to 23.4% (L_XS_, 50 OTUs) of OTUs but contributed more than 96.8% of total abundance. Cultivar-specific OTUs ranged from 36 (7.2%, L_XS_) to 75 (15%, H_Y1_), primarily consisting of RT, with total abundances below 0.35% in all cultivars (**Figure 1a**). Shannon diversity indices indicated high microbial diversity among cultivars, ranging from 2.95 ± 0.03 (L_FY_) to 3.23 ± 0.04 (H_Y1_), with significant differences (p < 0.05, one-way ANOVA, **Figure 1b**). All cultivars showed significantly higher Shannon diversity of rare taxa than intermediate and abundant taxa, while AT exhibited the lowest diversity (**Figure 1b**). Twelve bacterial phyla and 172 genera were identified, including 50 dominant genera with abundance > 0.1%. The number of dominant genera per cultivar ranged from 26 (L_FY_) to 35 (H_Y1_). Seventeen dominant genera were shared across all cultivars, including Methylobacterium, Xanthomonas, Buttiauxella, and Sphingomonas (**Figure 1c**).

#### 3.1.2 Community composition differences

**Figure 2:**
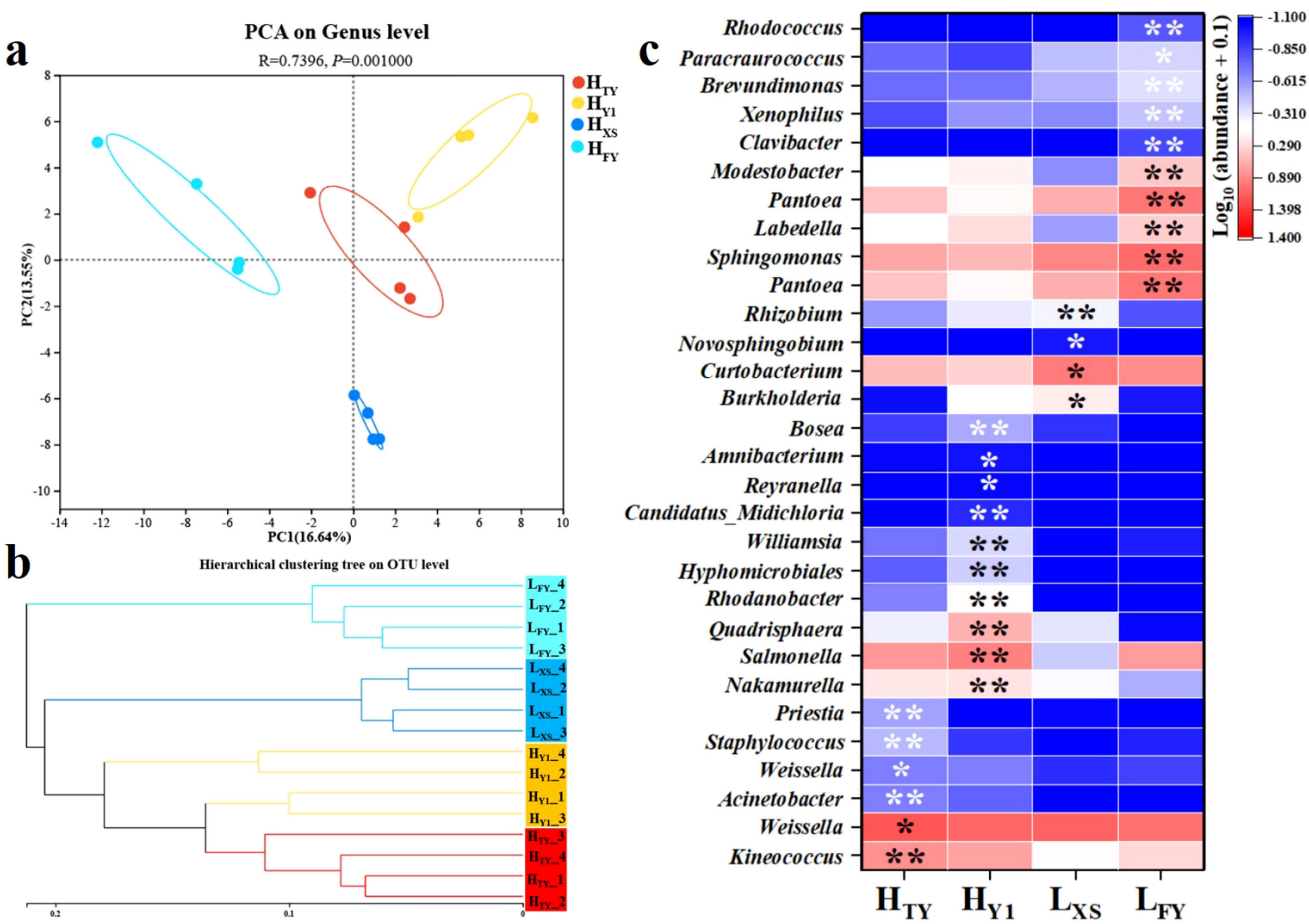
Principal component analysis (PCA) **(a)**, Hierarchical clustering **(b)**, and differential abundance analysis **(c)** of seed endophytic bacterial communities in High- (H_TY_, H_Y1_) and Low-NUE (L_FY_, L_XS_) rice varieties. Significance determined by Kruskal-Wallis H test: * p < 0.05, ** p < 0.01 (n = 4).

PCA revealed significant differences in seed endophytic bacterial communities among cultivars (p < 0.05, ANOSIM, **Figure 2a**). Hierarchical clustering showed that cultivars with the same NUE type clustered more closely, with H_TY_ and H_Y1_ (high-NUE) forming a distinct group (**Figure 2b**). Among the 172 identified genera, 60 (34.9%) showed significant abundance differences across cultivars (p < 0.05, Kruskal-Wallis H test). For example, Sphingomonas, which was dominant across all cultivars, was enriched in low-NUE cultivars (> 7.7% in L_XS_, 15.5% in L_FY_) but accounted for< 3.8% in both high-NUE cultivars (**Figure 2c**). Salmonella was dominant in H_TY_ (1.19%) and H_Y1_ (1.24%), but significantly reduced in L_FY_ (0.26%) and L_XS_ (0.76%). Conversely, Rhodanobacter was dominant in H_Y1_ (3.73%) but low (< 0.84%) in the other three cultivars. Additionally, some genera showed NUE-specific distributions, where Midichloria (0.002% in H_TY_, 0.016% in H_Y1_), Weissella (0.11% in H_TY_, 0.07% in H_Y1_), and Lichenibacterium (0.013% in H_TY_, 0.007% in H_Y1_) were found exclusively in high-NUE cultivars, whereas Sanguibacter (0.02% in L_FY_, 0.01% in L_XS_) was specific to low-NUE cultivars. (**Figure 2c**).

#### 3.1.3 Characteristics of microbial co-occurrence networks and indicator species

**Figure 3:**
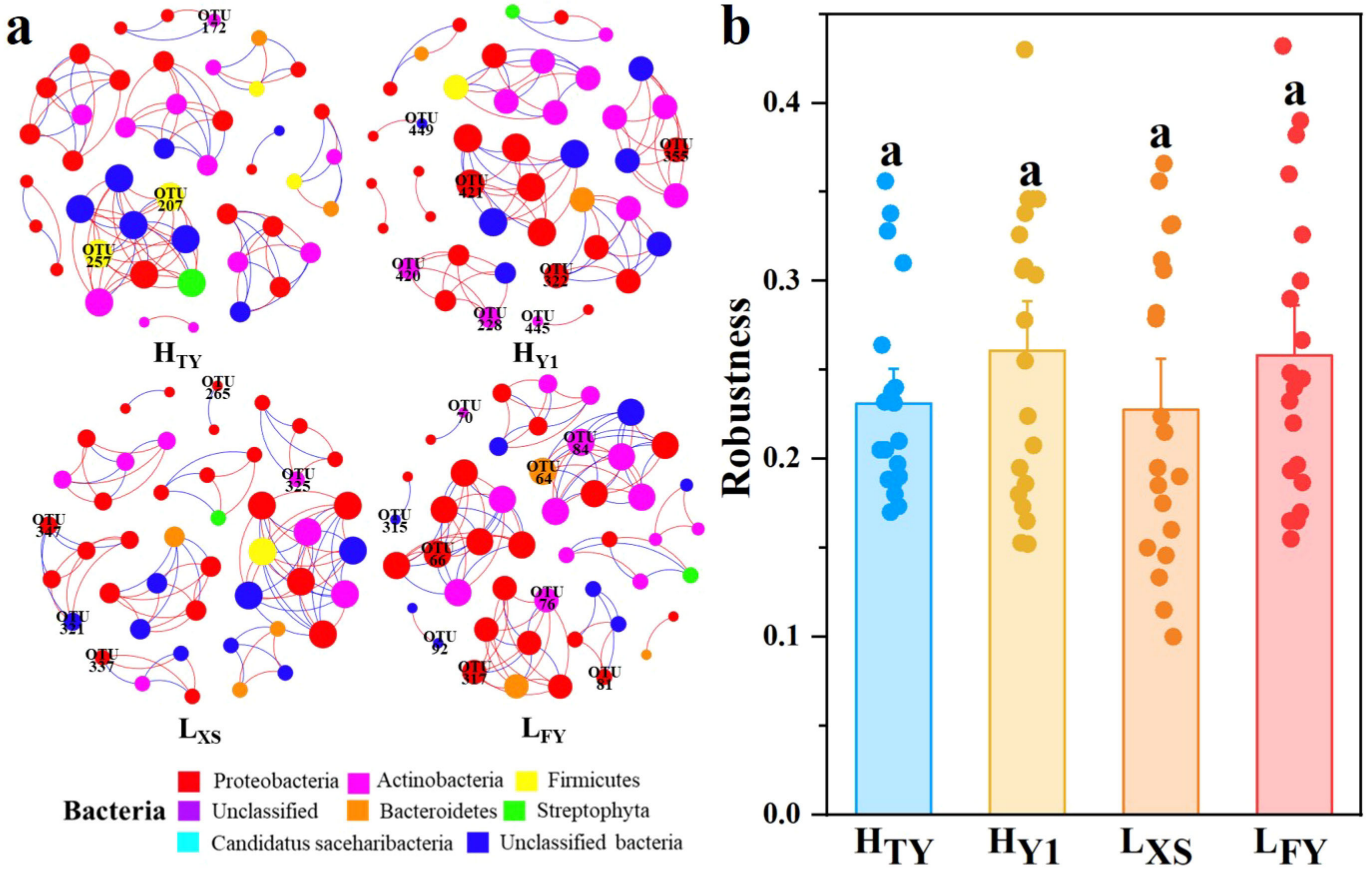
Co-occurrence network analysis **(a)** and microbial network robustness analysis **(b)** of seed endophytic bacteria in High- (H_TY_, H_Y1_) and Low- (L_FY_, L_XS_) NUE rice varieties. In the network, nodes are colored by phylum and node size reflects the degree. Red and blue edges indicate positive and negative correlations, respectively; OTU numbers correspond to indicator species identified in each cultivar.

Co-occurrence network analysis revealed similar topologies across four cultivars, with comparable numbers of edges and modularity indices, and predominantly positive correlations (**Figure 3a**; **Supplementary Table S2**). Several core OTUs, including Kineococcus (OTU286) and Pseudomonas (OTU310), served as shared network hubs across all cultivars, indicating structural similarity among networks. Network robustness indices ranged from 0.23 to 0.26 with no significant differences (p > 0.05, one-way ANOVA, **Figure 3b**). Indicator species analysis identified 76 cultivar-specific indicator OTUs (IndVal > 0.7, p < 0.05), none of which were shared among cultivars. The number of indicator OTUs ranged from 6 (H_TY_) to 27 (L_XS_). Specific indicators included Priestia (OTU207) for H_TY_, Actinomycetospora (OTU356) for H_Y1_, Aureimonas (OTU250) for L_XS_, and Clavibacter (OTU70) for L_FY_. Indicator OTUs for H_Y1_, L_FY_, and L_XS_ were primarily from Actinobacteria and Proteobacteria, while H_TY_’s indicators belonged mostly to Firmicutes (**Supplementary Table S3**).

#### 3.1.4 Functional prediction of bacterial communities

A total of 41 functional groups were annotated using the FAPROTAX database. Of these, 33 functions (80.5%) showed significant differences among cultivars (p < 0.05). Among the differential functions, 27.3% (9/33) were nitrogen-related (e.g., nitrogen fixation, nitrate reduction, urea decomposition), 21.2% were carbon cycling-related, and others involved sulfur cycling (9.09%) and pathogenicity (18.18%) (**Supplementary Figure S2**).

### 3.2 Isolation and growth-promoting potential of cultivable endophytes in high-NUE varieties

#### 3.2.1 Isolation and identification of cultivable endophytes

**Figure 4:**
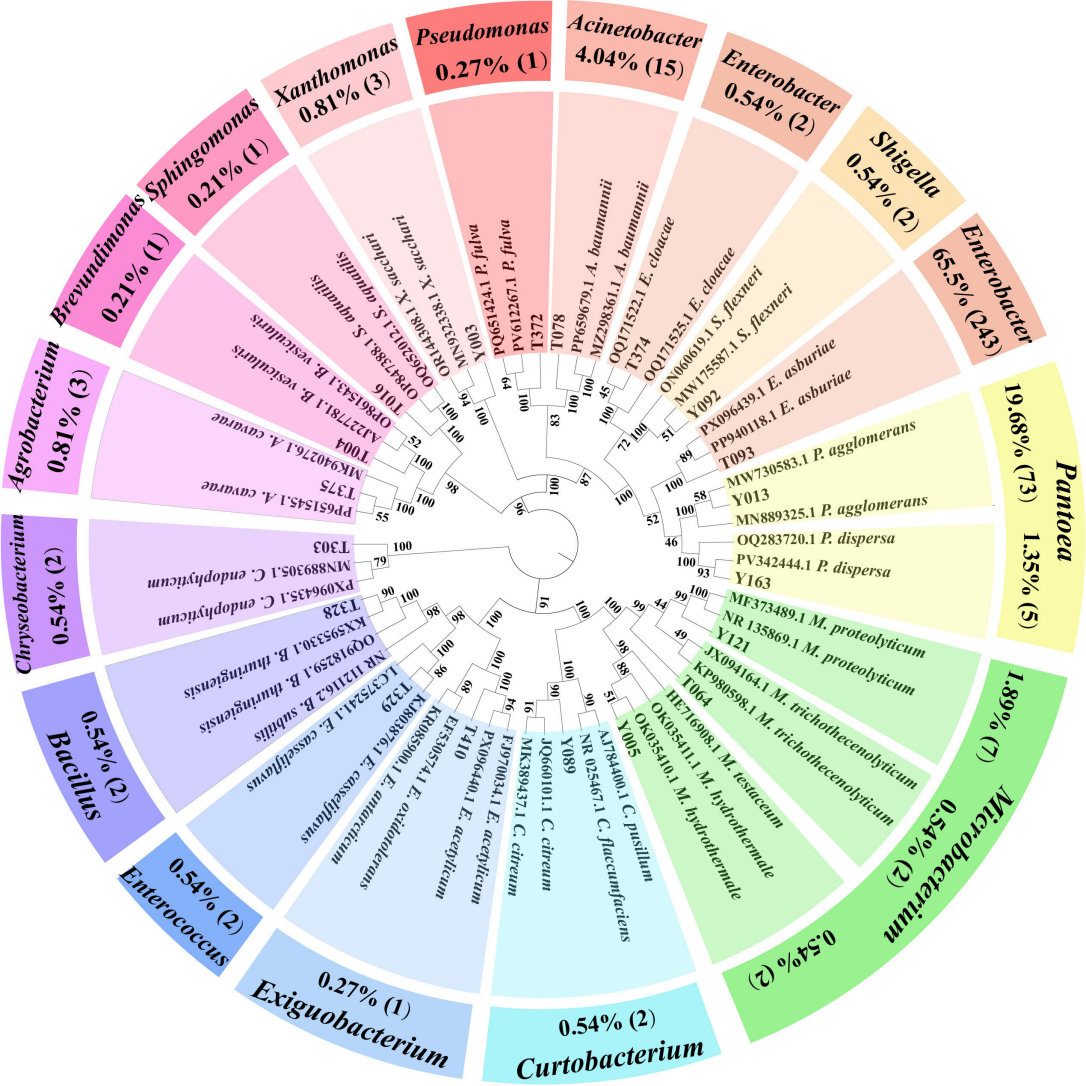
Neighbor-joining (NJ) phylogenetic tree based on the 16S rRNA sequences of 369 cultivable seed endophytic bacterial strains. The numbers above the nodes indicate bootstrap support values from 1,000 replicates. Colors in the inner circle distinguish bacterial genera, while the percentages in the outer ring show the isolation frequency. Arabic numerals in parentheses denote the number of isolates.

A total of 369 cultivable endophytes were isolated from seeds of H_TY_ and H_Y1_ cultivars. 16S rRNA gene sequencing revealed affiliation to 15 genera, with the most frequently isolated being Enterobacter (66.04%), Pantoea (21.03%), and Acinetobacter (4.04%). Based on frequency, diversity, and functional prediction, five representative strains were selected for the following experiments, including Bacillus thuringiensis T328, Xanthomonas sacchari Y003, Pantoea agglomerans Y013, Curtobacterium citreum Y089, and Pantoea dispersa Y163 (**Figure 4**; **Supplementary Table S5**).

#### 3.2.2 Growth-promoting traits of cultivable endophytes from high-NUE rice

**Figure 5:**
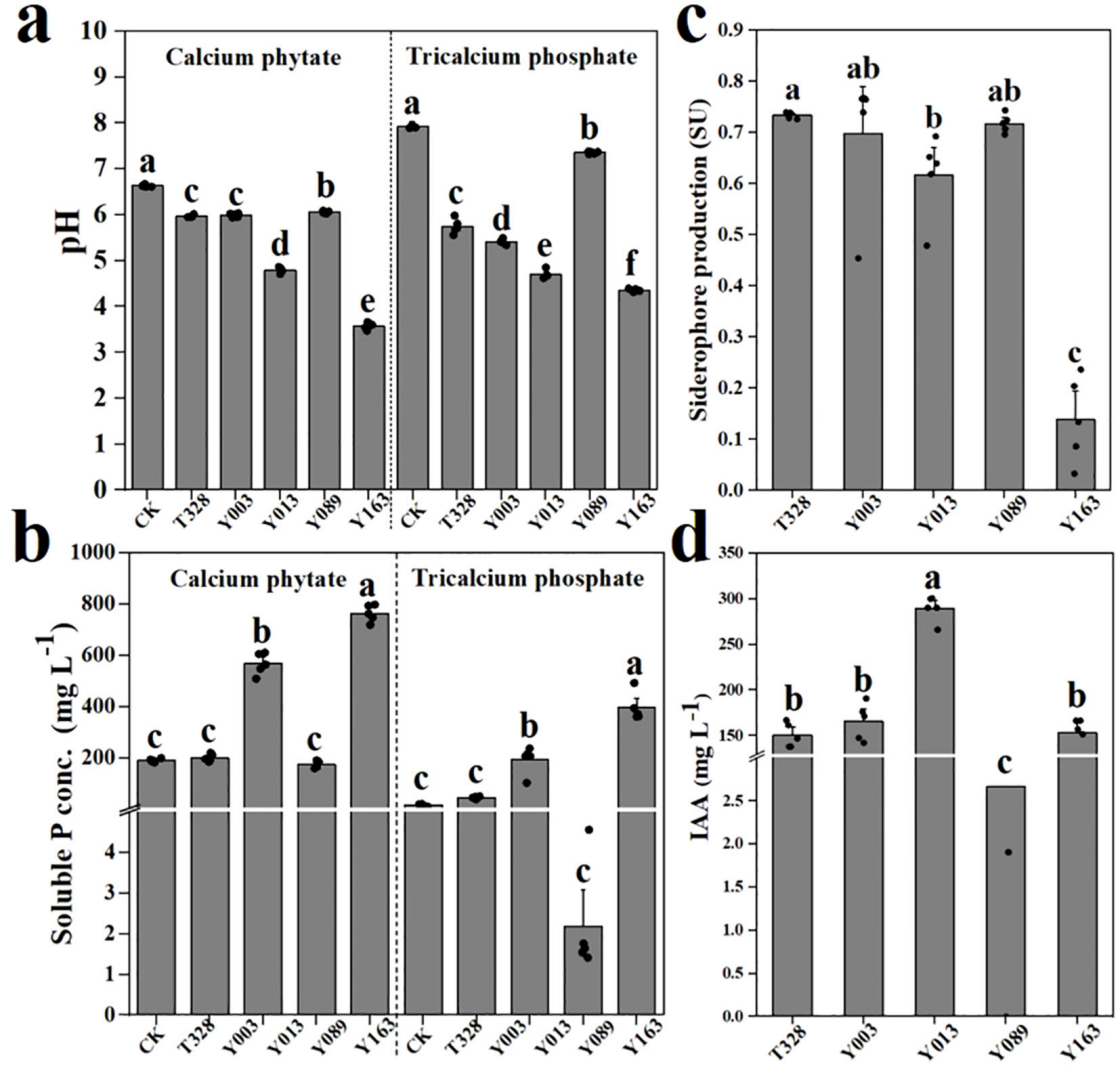
Plant growth-promoting (PGP) traits of five representative endophytes under *in vitro* conditions. **(a)** pH of the culture medium, **(b)** soluble phosphorus concentration in Pikovskaya (PVK) medium supplemented with calcium phytate and tricalcium phosphate as the sole phosphorus sources after 3 days of inoculation; **(c)** Siderophore production; and **(d)** IAA production. Data are means ± SE (n = 5); significance by one-way ANOVA (p < 0.05).

Five representative endophytes exhibited diverse plant growth-promoting traits, including phosphate solubilization, siderophore production, and IAA synthesis. Soluble phosphorus concentrations in fermentation broths ranged from 2.18 mg L^−1^ to 763.98 mg L^−1^. The siderophore units (SU) ranged from 0.139 to 0.733, and IAA concentrations varied between 2.67 mg L^−1^ and 289.38 mg L^−1^. Significant differences were observed in the dominant functions among strains. For example, P. dispersa Y163 exhibited the strongest phosphate solubilization ability, significantly higher than the other five strains, but its IAA production was lower than that of P. agglomerans Y013. Furthermore, the siderophore production index of P. dispersa Y163 was significantly lower than those of the other four strains, at only 0.139 (p < 0.05, one-way ANOVA, **Figure 5**).

### 3.3 Functional role of seed endophytes in enhancing rice growth and NUE

#### 3.3.1 Effects of seed endophytes on rice growth under different N levels

**Figure 6:**
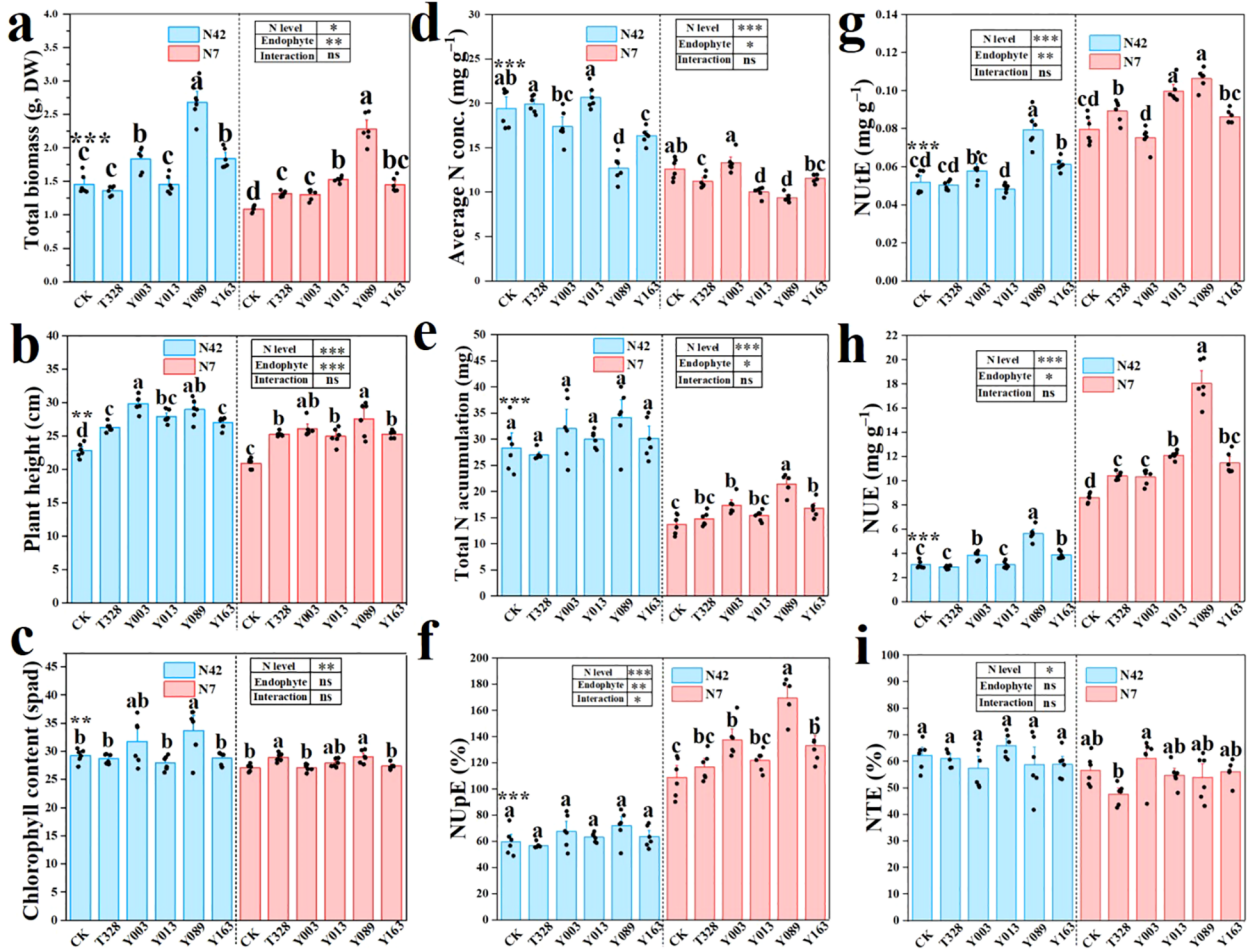
Effects of inoculation with five representative endophytes on rice total biomass **(a)**, plant height **(b)**, chlorophyll content **(c)**, average N concentration **(d)**, total N accumulation **(e)**, N uptake efficiency (NUpE) **(f)**, N utilization efficiency (NUtE) **(g)**, N use efficiency **(h)**, and N transport efficiency (NTE) **(i)**, under normal nitrogen (N42) and low nitrogen (N7) treatments. Mean ± SE (n = 6). Different lowercase letters indicate significant differences at the same N level (p < 0.05, one-way ANOVA).

Compared with the uninoculated control, inoculation with five representative endophytes enhanced rice growth, including biomass, plant height, chlorophyll content, tiller number, number of fresh leaves, and root length, while reducing the number of withered leaves across treatments (**Figure 6**; **Supplementary Figure S3**). However, the inoculation effect was influenced by the combined factors of bacterial strain type and nitrogen level. Under normal nitrogen conditions (N42), most strains inoculation—particularly C. citreum Y089, P. dispersa Y163 and X. sacchari Y003—significantly increased shoot dry biomass (p < 0.05, one-way ANOVA). In contrast, B. thuringiensis T328 and P. agglomerans Y013 showed no significant effect (p > 0.05, one-way ANOVA). Interestingly, under low nitrogen conditions (N7), all strains promoted rice growth significantly (p < 0.05, one-way ANOVA, **Figure 6a**). Among the five representative endophytes, C. citreum Y089 consistently exhibited superior growth-promoting effects under both normal and low nitrogen conditions, indicating its potential as the most effective plant growth-promoting strain (**Figure 6a**).

#### 3.3.2 Seed endophytes enhance nitrogen accumulation and NUE in rice

Compared to the uninoculated control, inoculation with the five representative endophytes did not significantly increase the average N concentration in rice under either nitrogen regime and even showed a general decreasing trend (**Figure 6d**). In contrast, total N accumulation per plant was significantly improved by bacterial inoculation in all treatments. Notably, inoculation with C. citreum Y089 increased total N accumulation by 20.5% under N42 and 55.7% under N7 conditions, respectively (**Figure 6e**). Correspondingly, inoculation with representative endophytes also enhanced N uptake efficiency (NUpE), nitrogen utilization efficiency (NUtE), and nitrogen use efficiency (NUE) of rice, but did not affect N transport efficiency (NTE). C. citreum Y089 showing the most pronounced effect: its NUtE increased significantly by 53.1% under N42 and 33.6% under N7. Meanwhile, its NUE was markedly enhanced by 0.84-fold under N42 and 1.1-fold under N7 compared with the CK group (p < 0.05, one-way ANOVA) (**Figure 6**). It is worth noting that the effect of bacterial inoculation on NUE was influenced by both bacterial strain and nitrogen availability (**Figure 6i**). For example, B. thuringiensis T328 and P. agglomerans Y013 did not significantly improve NUE under normal nitrogen conditions, but under N7 they increased NUE by 21.17% and 20.22%, respectively (p < 0.05, one-way ANOVA, **Figure 6h**).

## 4 Discussion

Using both culture-dependent and culture-independent approaches, we detected a rich and diverse array of endophytic bacterial taxa in the seeds of all four rice cultivars examined, with Shannon diversity indices ranging from 2.99 to 3.23. These findings are consistent with those of Deckert et al. (2019), who identified highly diverse seed endophytic communities in two representative gymnosperms, Pinus edulis and P. ponderosa, including four mycorrhizal fungal genera (e.g., Gymnopilus). A meta-analysis by Simonin et al. (2022) encompassing seed microbiota from 50 plant species further confirmed the widespread high diversity of microbial communities residing within plant seeds. Together, these findings support the view that plant seeds represent a rich reservoir of endophytic microbes and form an integral part of the plant microbiome (Nelson, 2018).

We found that the composition of seed endophytic bacterial communities varied significantly among different rice cultivars. Such variation is commonly observed not only across different plant species but also among genotypes within the same species. Simonin et al. (2022) reported substantial diversity in seed endophytic communities across 50 plant species, with taxonomic richness ranging from a single taxon to several thousand, averaging 95 taxa per seed (34 prokaryotic and 61 fungal taxa). Similarly, Wang et al. (2023a) observed pronounced differences in seed endophytic bacterial composition among 11 genetically correlated hybrid rice seeds with different rice blast resistance levels, even among hybrids with similar genetic constitutions, such as those sharing the same female parent but different male parents, and vice versa. It is widely accepted that the composition of seed-associated endophytes results from the interplay of multiple factors, with host genotype being one of the most critical drivers (da Silva et al., 2014). In a more recent study, Lobato et al. (2024) compared the impact of plant genotype, domestication, and breeding on endophytic bacterial communities in the seeds of 46 Cannabis genotypes, including early domestication genotypes obtained through natural and artificial selection, as well as modern genotypes obtained through artificial selection. The study found that the heterogeneous genetic background of Cannabis influences its seed microbiome. In addition to genetic factors, abiotic environmental variables such as soil type, climate, moisture, and nutrient availability also contribute to shaping the seed microbiome (Durán et al., 2022), suggesting the combined influence of host genotype, environmental factors, and microbial ecological interactions (Franić et al., 2020).

Among the four rice cultivars analyzed, flexible rare taxa (RT) played a dominant role in shaping both the diversity and composition of the seed endophytic microbiome. RT exhibited the highest OTU richness and Shannon diversity despite their low relative abundance. This pattern aligns with observations from diverse soil ecosystems, where rare taxa frequently contribute disproportionately to overall diversity and serve as key regulators of microbial compositional variation (Yang et al., 2025). In contrast to the high variability of RT, a subset of abundant taxa (AT, > 93% of total relative abundance) was consistently detected in the seeds of all four rice cultivars, indicating broad host adaptability. For example, genera such as Sphingomonas, Methylobacterium, and Pantoea are not only prevalent in major crops like rice, maize, and wheat but also widely distributed in woody or leguminous hosts (Suman et al., 2020; Simonin et al., 2022). Notably, these taxa can also persist under extreme environmental stresses, including drought, salinity, and heavy metal contamination (Asaf et al., 2017; Li et al., 2023; Tai et al., 2024). Importantly, these abundant and shared core taxa also showed strong genetic conservation across various rice cultivars and were similarly conserved across multiple hybrid generations and geographical regions (Walitang et al., 2019). Collectively, these findings suggest that vertical transmission and genetic conservation of core seed endophytes contribute to their long-term association with host plants and play a critical role in plant–microbiome co-evolution.

We also observed that these core taxa frequently occupied central positions within the microbial co-occurrence networks, suggesting their critical role in maintaining community stability (Jiao et al., 2019; Li et al., 2021). In particular, core microbes contributing to essential host functions—such as nitrogen fixation or disease resistance—may be selectively retained by the host to ensure the stability and integrity of microbiome function (Walitang et al., 2019). In parallel, rare taxa also contribute significantly to ecosystem functioning. On one hand, they harbor a highly diverse repertoire of functional genes, serving as a vast gene reservoir within the microbial community (Chen et al., 2020; Zhou et al., 2022). The functional diversity among RT increases microbial functional redundancy, providing “backup” capacities that buffer the community against external disturbances and thereby enhance ecological stability and resilience (Kang et al., 2015; Louca et al., 2018). On the other hand, rare taxa serve as a “microbial seed bank” within the community. Conditionally rare taxa can dynamically shift between rare and abundant states depending on environmental conditions, thereby influencing both the structure and function of the microbial community (Lynch and Neufeld, 2015; Hu et al., 2023). Although low in abundance, rare taxa exert disproportionate influence on microbial community diversity and functional capacity. They are now recognized as essential contributors to the stability and resilience of microbial ecosystems (Lynch and Neufeld, 2015; Jousset et al., 2017). Taken together, the conserved core taxa and the highly variable rare taxa confer a “dual-stability” to the seed microbiome, characterized by a stable core microbiome and a flexible rare biosphere. This duality may reflect an evolutionary strategy for microbial adaptation to host selection.

We found that representative endophytes in rice exhibited diverse and functionally redundant PGP traits. However, these strains displayed differentiated strengths in their functional profiles, indicating a degree of functional complementarity. Such functional diversity and redundancy are common features among seed-associated endophytes in various plant species. For instance, numerous endophytic bacteria isolated from rice and tomato seeds have been shown to produce IAA, siderophores, and solubilize essential minerals such as phosphorus and potassium (Jana et al., 2023; Roy et al., 2024). Functional redundancy and complementarity among microbial PGP traits are key mechanisms supporting host growth and adaptive capacity. On one hand, functional redundancy ensures the stability of host-associated benefits under variable environmental conditions by mitigating the loss of any single strain (Vandenkoornhuyse et al., 2015; Wang et al., 2023b). On the other hand, complementary interactions among functionally distinct strains can enhance the host’s functional diversity, thereby promoting greater ecological adaptability (Singh et al., 2015; Finkel et al., 2017). For example, compared to single-strain inoculation, Paenibacillus sp. B1 acidified the rhizosphere pH through organic acid secretion, thereby enhancing the high-level expression of the nifH gene in the co-inoculated strain Paenibacillus beijingensis and activating soil nitrogenase activity. This co-inoculation significantly promoted wheat growth, particularly increasing root nitrogen and phosphorus accumulation in wheat roots by 27% and 63%, respectively (Li et al., 2020). Therefore, when assessing the functional roles of seed microbiota, it is important to consider both functional redundancy and complementarity among strains. By following these principles, the rational design of effective functional microbial communities can be achieved, laying the theoretical groundwork for the practical use of seed endophytes in agriculture (Northen et al., 2024; Zhu et al., 2025).

The five representative endophytes significantly enhanced rice growth, nitrogen accumulation, and NUE, likely through multiple synergistic mechanisms. Firstly, nitrogen-fixing seed endophytes can effectively convert atmospheric nitrogen (N_2_) into plant-available forms, such as ammonium (NH_4_^+^), thereby increasing the nitrogen supply to the host (Huang et al., 2023). Secondly, numerous endophytic bacteria can modulate host nitrogen metabolism and contribute to nitrogen cycling in the rhizosphere (Liang and Bowatte, 2022). For example, Epichloë gansuensis significantly enhances the activities of nitrate reductase, nitrite reductase, and glutamine synthetase in Achnatherum inebrians, thereby accelerating the conversion of NO_3_^−4^ to organic nitrogen and improving NUE (Wang et al., 2018). Thirdly, endophytic fungi can modify and improve the rhizosphere microenvironments, creating favorable niches for beneficial microbes, including nitrogen fixers (Li et al., 2020; Kour et al., 2021). For example, seed endophytes such as Fictibacillus rigui and Priestia aryabhattai accelerate the reduction of Fe^3+^ to Fe^2+^ increasing electron availability for the DNRA pathway. This, in turn, selectively recruits DNRA-performing Clostridium spp., strengthening nitrogen-conserving processes and ultimately enhancing rice NUE (Liu et al., 2025). Moreover,many seed endophytes, such as Burkholderia vietnamiensis RS1, can produce plant hormones like IAA, which stimulate root cell division and elongation. This promotes the formation of lateral roots and root hairs, ultimately enhancing nitrogen uptake (Shinjo et al., 2022). Collectively, these findings underscore the critical role of seed endophytes in facilitating nitrogen acquisition and translocation in host plants.

However, we also observed that the growth-promoting effects of seed endophytes are co-determined by bacterial strain and nitrogen conditions. Firstly, different bacterial strains can trigger distinct host response mechanisms, leading to variable growth outcomes. For instance, multiple strains of Paenibacillus inoculated into wheat, cucumber, and tomato exhibited markedly different PGP effects by suppressing ethylene levels and promoting root development (Liu et al., 2019). Interestingly, even the same bacterial strain may exhibit different PGP effects under varying environmental stress conditions. For example, under low-nitrogen conditions, inoculation with Bradyrhizobium japonicum enhanced soybean nitrogen accumulation by approximately 40% through symbiotic nitrogen fixation. However, under high-nitrogen conditions, soybean plants favored the energy-efficient strategy of direct nitrogen uptake over the energetically costly process of symbiosis. As a result, B. japonicum inoculation had no significant effect on nitrogen accumulation in high-nitrogen environments (Perkowski et al., 2024). These findings highlight the importance of considering both microbial and environmental factors in the practical application of seed endophytes. Optimal outcomes depend not only on selecting the appropriate bacterial strains but also on understanding their functional traits in relation to specific environmental conditions.

## 5 Conclusion

This study compared the composition of seed endophytic bacterial communities between high- and low-NUE rice cultivars. The results revealed that rice seeds harbor highly diverse endophytic bacterial communities, with significant differences in community structure among cultivars. Despite these differences, all four cultivars exhibited a common dual pattern: rare taxa serve as key drivers of community diversity and compositional variation, while core taxa display strong genetic conservation across cultivars. The representative seed endophytic bacteria possessed diverse, functionally redundant, and complementary PGP traits, which significantly enhanced rice growth and NUE. However, the magnitude and consistency of their beneficial effects were modulated by both the bacterial strain and environmental nitrogen availability. Overall, this study offers important theoretical insights into the diversity and functional potential of the rice seed microbiome, providing a solid foundation for developing and applying seed endophytes as microbial resources to enhance nitrogen use efficiency in rice cultivation.

## Data Availability

The original contributions presented in the study are included in the article/**Supplementary Material**. Further inquiries can be directed to the corresponding authors.
