# Supplementary material for: Nature’s pre-installed helpers: diverse seed endophytes enhance rice nitrogen use efficiency
Source: Front Plant Sci. 2026 Jan 20;16:1709648. doi: 10.3389/fpls.2025.1709648 (PMC12864439; doi:10.3389/fpls.2025.1709648)
Supplement: Supplementary file 1 [file DataSheet1.docx]

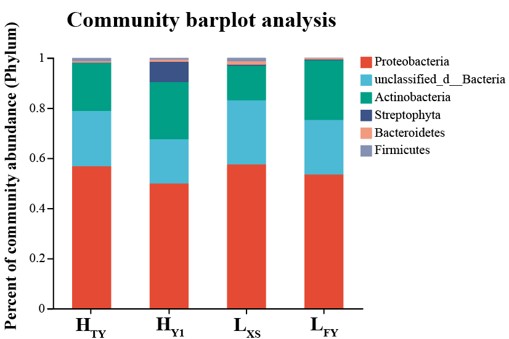
**Figure S1.** Species composition analysis of seed endophytic bacterial communities in high - (H_TY_, H_Y1_) and low- (L_XS_, L_FY_) nitrogen use efficiency rice varieties at phylum level (*n* = 4).


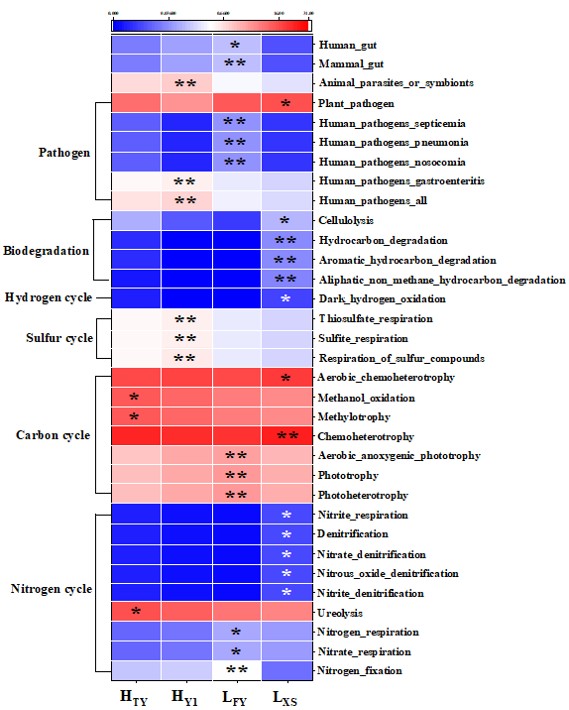
**Figure S2.** Ecological function prediction of species or genera of seed endophytic bacteria from High- (H_TY_, H_Y1_) and Low- (L_FY_, L_XS_) nitrogen use efficiency rice varieties based on the FAPROTAX database, with intergroup difference tests of functional prediction results performed using the Wilcoxon rank-sum test (*n* = 4).


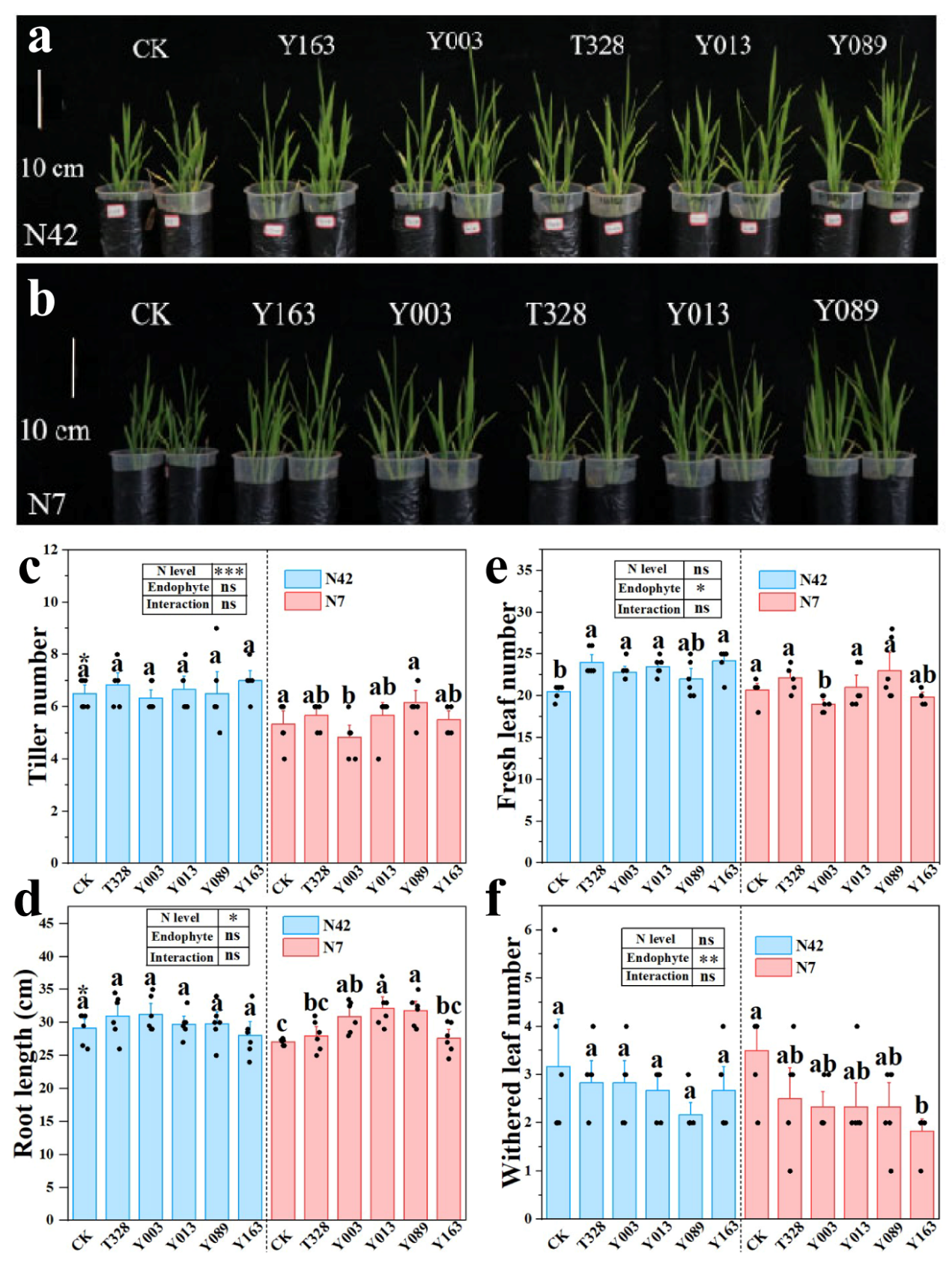


**Fi****gure S3.** Effects of inoculation with five endophytic bacteria strains on rice growth: growth status (a, b), tiller number (c), root length (d), fresh leaf number (e), and withered leaf number (f) under normal nitrogen (N42) and low nitrogen (N7) treatments. Mean ± SE (*n* = 6). Different lowercase letters indicate significant differences at the same nitrogen level (*p* < 0.05, one-way ANOVA).

**Table S1.** Nitrogen use efficiency (NUE) characteristics of the tested seven rice varieties

| **Rice varieties** | **Rice type** | **Parents** | | **References** |
| --- | --- | --- | --- | --- |
|  |  | **Female** | **Male** |  |
| **High- NUE varieties** | | | | |
| Tianyouhuazhan (H_TY_) | *Indica* hybrid rice | Tianfeng A | Huazhan | Feng et al., 2014; Hu et al., 2019 |
| Y Liangyou 1# (H_Y1_) | *Indica* hybrid rice | Y58S | 93-11 | Cui et al., 2010 |
| **Low- NUE varieties** | | | | |
| Xiushui 134# (L_XS_) | *Japonica* conventional rice | Bing95-59//Ce12/RHT | [Bing 03-123](https://www.ricedata.cn/variety/varis/604788.htm) | Feng et al., 2014 |
| Fuyuan 4# (L_FY_) | *Japonica* conventional rice | [31116S+30301S](https://www.ricedata.cn/variety/varis/0+0+0+0.htm)  [5047S+4018S](https://www.ricedata.cn/variety/varis/0+0+0+0.htm) | [Chaochan 1#,](https://www.ricedata.cn/variety/varis/601494+605338.htm)  [Chaochan 2#](https://www.ricedata.cn/variety/varis/601494+605338.htm) | An et al., 2014 |

**Table S2.** Topological properties of endophytic bacterial co-occurrence networks in the seeds of High- (H_TY_, H_Y1_) and Low- (L_FY_, L_XS_) nitrogen use efficiency rice varieties (*n* = 6).

| **Topological structure characteristics** | **Variety** | | | |
| --- | --- | --- | --- | --- |
|  | **H_TY_** | **H_Y1_** | **L_XS_** | **L_FY_** |
| Nodes | 45 | 44 | 45 | 47 |
| Edges | 101 | 86 | 97 | 106 |
| Avg. degree | 4.489 | 3.909 | 4.311 | 4.511 |
| Avg. path length | 1 | 1 | 1 | 1 |
| Modularity | 0.798 | 0.833 | 0.802 | 0.805 |
| Positive correlation ratio (%) | 57.43 | 69.77 | 53.61 | 53.77 |
| Negative correlation ratio (%) | 42.57 | 30.23 | 46.39 | 46.23 |

**Table S3.** Information on the top 20 core nodes in co-occurrence networks of seed endophytic bacterial communities in High - (H_TY_, H_Y1_) and Low - (L_XS_, L_FY_) nitrogen use efficiency rice varieties.

| **Phylum** | **Genus** | **Variety** | | | |
| --- | --- | --- | --- | --- | --- |
|  |  | **H_TY_** | **H_Y1_** | **L_XS_** | **L_FY_** |
| unclassified Bacteria | unclassified Bacteria | OTU351 | OTU351 | OTU334 | OTU351 |
|  |  | OTU304 | OTU304 | OTU304 |  |
|  |  | OTU296 | OTU296 | OTU326 |  |
|  |  | OTU345 | OTU345 | OTU330 |  |
|  |  |  | OTU249 |  |  |
| Actinobacteria | *Kineococcus* | OTU286 | OTU286 | OTU286 | OTU286 |
|  | *Microbacterium* | OTU268 | OTU268 | OTU268 |  |
|  | *Labedella* | OTU302 | OTU302 |  | OTU302 |
|  | *Curtobacterium* | OTU261 |  | OTU261 | OTU261 |
|  | *Quadrisphaera* |  | OTU276 | OTU276 | OTU276 |
|  | *Microbacterium* |  | OTU278 | OTU278 |  |
|  | *Modestobacter* |  | OTU258 |  | OTU258 |
|  | *Gordonia* | OTU295 |  |  |  |
|  | *Salana* |  |  |  | OTU84 |
| Bacteroidetes | *Chryseobacterium* |  |  | OTU289 |  |
|  | *Hymenobacter* |  |  |  | OTU64 |
| Firmicutes | *Paenibacillus* |  | OTU293 | OTU293 |  |
|  | *Priestia* | OTU207 |  |  |  |
|  | *Staphylococcus* | OTU275 |  |  |  |
| Proteobacteria | *Pseudomonas* | OTU310 | OTU310 | OTU310 | OTU310 |
|  | *Methylorubrum* | OTU324 | OTU324 |  | OTU324 |
|  | *Xanthomonas* | OTU257 | OTU257 |  | OTU257 |
|  | *Methylobacterium* | OTU287 |  | OTU287 | OTU287 |
|  | *Buttiauxella* | OTU364 |  | OTU364 |  |
|  | *Pantoea* | OTU320 |  |  | OTU320 |
|  | *Salmonella* |  | OTU426 |  | OTU426 |
|  | *Sphingomonas* |  | OTU328 | OTU328 |  |
|  | *Methylobacterium* |  |  | OTU266 | OTU266 |
|  | *Paracraurococcus* |  |  | OTU271 | OTU271 |
|  | *Methylobacterium* | OTU305 |  |  |  |
|  | *Pantoea* | OTU309 |  |  |  |
|  | *Aureimonas* |  | OTU212 |  |  |
|  | *Devosia* |  | OTU421 |  |  |
|  | *Rhodanobacter* |  | OTU322 |  |  |
|  | *Herbaspirillum* |  |  | OTU353 |  |
|  | *Methylobacterium* |  |  | OTU291 |  |
|  | *Xenophilus* |  |  | OTU333 |  |
|  | *Achromobacter* |  |  |  | OTU299 |
|  | *Methylobacterium* |  |  |  | OTU169 |
|  | *Methylobacterium* |  |  |  | OTU317 |
|  | *Pantoea* |  |  |  | OTU66 |
| Streptophyta | *Oryza* | OTU262 |  |  |  |

**Figure S4.** Indicator species analysis of seed endophytic bacteria in High- (H_TY_, H_Y1_) and Low- (L_FY_, L_XS_) nitrogen use efficiency rice varieties (*n* = 4).

| **Variety** | **OTU** | **Phylum** | **Genus** | **Indval** | ***P* value** |
| --- | --- | --- | --- | --- | --- |
| **H_TY_** | OTU207 | Firmicutes | *Priestia* | 0.97 | 0.003 |
|  | OTU125 | unclassified | unclassified | 0.94 | 0.009 |
|  | OTU275 | Firmicutes | *Staphylococcus* | 0.88 | 0.006 |
|  | OTU95 | Proteobacteria | *Methylobacterium* | 0.76 | 0.043 |
|  | OTU172 | Actinobacteria | *Streptomyces* | 0.74 | 0.046 |
|  | OTU442 | unclassified | unclassified | 0.73 | 0.049 |
| **H_Y1_** | OTU460 | unclassified | unclassified | 1.00 | 0.002 |
|  | OTU441 | unclassified | unclassified | 1.00 | 0.003 |
|  | OTU438 | unclassified | unclassified | 0.99 | 0.001 |
|  | OTU449 | unclassified | unclassified | 0.97 | 0.004 |
|  | OTU432 | unclassified | unclassified | 0.96 | 0.003 |
|  | OTU356 | Actinobacteria | *Actinomycetospora* | 0.93 | 0.007 |
|  | OTU435 | unclassified | unclassified | 0.91 | 0.002 |
|  | OTU451 | Bacteroidetes | *Siphonobacter* | 0.90 | 0.003 |
|  | OTU420 | Actinobacteria | *Nakamurella* | 0.89 | 0.003 |
|  | OTU322 | Proteobacteria | *Rhodanobacter* | 0.82 | 0.004 |
|  | OTU416 | unclassified | unclassified | 0.82 | 0.007 |
|  | OTU445 | Proteobacteria | unclassified | 0.81 | 0.001 |
|  | OTU355 | Proteobacteria | *Bosea* | 0.79 | 0.002 |
|  | OTU319 | unclassified | unclassified | 0.79 | 0.014 |
|  | OTU430 | Proteobacteria | *Moraxella* | 0.79 | 0.01 |
|  | OTU399 | unclassified | unclassified | 0.78 | 0.007 |
|  | OTU228 | Actinobacteria | *Williamsia* | 0.76 | 0.003 |
|  | OTU203 | unclassified | unclassified | 0.76 | 0.005 |
|  | OTU459 | Proteobacteria | unclassified | 0.76 | 0.013 |
|  | OTU446 | unclassified | unclassified | 0.75 | 0.019 |
|  | OTU444 | Firmicutes | *Paenibacillus* | 0.75 | 0.021 |
|  | OTU417 | Bacteroidetes | *Spirosoma* | 0.75 | 0.028 |
|  | OTU423 | Firmicutes | *Clostridium* | 0.75 | 0.028 |
|  | OTU382 | Proteobacteria | *Sphingomonas* | 0.75 | 0.033 |
|  | OTU421 | Proteobacteria | Devosia | 0.73 | 0.005 |
|  | OTU246 | Actinobacteria | *Patulibacter* | 0.72 | 0.018 |
|  | OTU436 | unclassified | unclassified | 0.71 | 0.017 |
| **L_XS_** | OTU250 | Proteobacteria | *Aureimonas* | 1.00 | 0.001 |
|  | OTU354 | unclassified | unclassified | 1.00 | 0.004 |
|  | OTU348 | Bacteroidetes | *Spirosoma* | 1.00 | 0.007 |
|  | OTU323 | Proteobacteria | *Aureimonas* | 0.96 | 0.005 |
|  | OTU325 | Actinobacteria | *Microbacterium* | 0.95 | 0.002 |
|  | OTU321 | unclassified | unclassified | 0.94 | 0.002 |
|  | OTU347 | Proteobacteria | *Sphingomonas* | 0.94 | 0.005 |
|  | OTU337 | Proteobacteria | *Burkholderia* | 0.93 | 0.003 |
|  | OTU363 | Proteobacteria | *Ochrobactrum* | 0.92 | 0.002 |
|  | OTU368 | unclassified | unclassified | 0.92 | 0.006 |
|  | OTU248 | unclassified | unclassified | 0.91 | 0.015 |
|  | OTU265 | Proteobacteria | *Sphingomonas* | 0.90 | 0.001 |
|  | OTU222 | unclassified | unclassified | 0.88 | 0.002 |
|  | OTU274 | Proteobacteria | *Methylobacterium* | 0.83 | 0.013 |
|  | OTU263 | unclassified | unclassified | 0.80 | 0.009 |
|  | OTU154 | Proteobacteria | *Pseudoxanthomonas* | 0.78 | 0.002 |
|  | OTU346 | Firmicutes | *Saccharibacillus* | 0.76 | 0.009 |
|  | OTU283 | unclassified | unclassified | 0.76 | 0.003 |
|  | OTU220 | Bacteroidetes | *Mucilaginibacter* | 0.75 | 0.025 |
|  | OTU217 | Proteobacteria | *Methylobacterium* | 0.75 | 0.028 |
|  | OTU290 | Actinobacteria | *Leucobacter* | 0.75 | 0.028 |
|  | OTU357 | Proteobacteria | *Novosphingobium* | 0.75 | 0.032 |
|  | OTU372 | Proteobacteria | *Methylobacterium* | 0.75 | 0.035 |
|  | OTU259 | Proteobacteria | *Klebsiella* | 0.74 | 0.002 |
|  | OTU273 | Proteobacteria | *Sphingomonas* | 0.72 | 0.003 |
|  | OTU272 | Proteobacteria | *Methylobacterium* | 0.71 | 0.004 |
| **L_FY_** | OTU70 | Actinobacteria | *Clavibacter* | 1.00 | 0.002 |
|  | OTU76 | Actinobacteria | *Arthrobacter* | 1.00 | 0.004 |
|  | OTU94 | Actinobacteria | *Rhodococcus* | 0.98 | 0.004 |
|  | OTU315 | unclassified | *unclassified* | 0.98 | 0.004 |
|  | OTU81 | Proteobacteria | *Massilia* | 0.97 | 0.006 |
|  | OTU64 | Bacteroidetes | *Hymenobacter* | 0.91 | 0.004 |
|  | OTU318 | Proteobacteria | *Roseomonas* | 0.91 | 0.005 |
|  | OTU66 | Proteobacteria | *Pantoea* | 0.88 | 0.002 |
|  | OTU91 | Proteobacteria | *Rhizobium* | 0.86 | 0.006 |
|  | OTU84 | Actinobacteria | *Salana* | 0.85 | 0.004 |
|  | OTU77 | Proteobacteria | Paracoccus | 0.85 | 0.006 |
|  | OTU92 | unclassified | unclassified | 0.82 | 0.004 |
|  | OTU56 | unclassified | unclassified | 0.79 | 0.009 |
|  | OTU33 | Proteobacteria | *Methylobacterium* | 0.75 | 0.034 |
|  | OTU25 | Bacteroidetes | *Chryseobacterium* | 0.75 | 0.044 |
|  | OTU317 | Proteobacteria | *Methylobacterium* | 0.74 | 0.005 |
|  | OTU85 | Bacteroidetes | *Chryseobacterium* | 0.74 | 0.012 |

**Table S5**. Diversity of culturable seed endophytic bacteria in high nitrogen use efficiency rice varieties (H_TY_, H_Y1_)

| **Strains** | **Isolation frequency (strain Number)** | **Accession No.** |  | **Best matched in High-throughput sequencing** | | |  | **Top-hit NCBI sequence blast*** | | |
| --- | --- | --- | --- | --- | --- | --- | --- | --- | --- | --- |
|  |  |  |  | **OTU** | **Abundance** | **Iden. (%)** |  | **Accession No.** | **Iden. (%)** | **Taxonomy** |
| ***Acinetobacter*** |  |  |  |  |  |  |  |  |  |  |
| T078 | 4.07% (15) | PX096431.1 |  | OTU288 | 0.079% | 98.28 |  | MZ298361.1 | 100 | *A. baumannii* |
| ***Agrobacterium*** |  |  |  |  |  |  |  |  |  |  |
| T375 | 0.81% (3) | PX096432.1 |  | OTU479 | 0.003% | 100 |  | PP651545.1 | 100 | *A.* *cavarae* |
| ***Bacillus*** |  |  |  |  |  |  |  |  |  |  |
| T328 | 0.54% (2) | PX096433.1 |  | OTU227 | 0.003% | 100 |  | OQ918259.1 | 100 | *B. thuringiensis* |
| ***Brevundimonas*** |  |  |  |  |  |  |  |  |  |  |
| T004 | 0.27% (1) | PX096434.1 |  | OTU359 | 0.086% | 97.76 |  | OP861543.1 | 99.69 | *B. vesicularis* |
| ***Chryseobacterium*** |  |  |  |  |  |  |  |  |  |  |
| T303 | 0.54% (2) | PX096435.1 |  | OTU308 | 0.003% | 97.50 |  | MN889305.1 | 100 | *C. endophyticum* |
| ***Curtobacterium*** |  |  |  |  |  |  |  |  |  |  |
| Y089 | 0.54% (2) | PX096436.1 |  | OTU261 | 2.654% | 100 |  | MK389437.1 | 100 | *C. citreum* |
| ***Enterococcus*** |  |  |  |  |  |  |  |  |  |  |
| T329 | 0.54% (2) | PX096437.1 |  | OTU213 | 0.057% | 100 |  | LC375241.1 | 99.86 | *E. casseliflavus* |
| ***Enterobacter*** |  |  |  |  |  |  |  |  |  |  |
| T374 | 0.54% (2) | PX096438.1 |  | OTU349 | 0.031% | 100 |  | OQ171522.1 | 99.85 | *E. cloacae* |
| T093 | 65.85% (243) | PX096439.1 |  | OTU349 | 1.958% | 100 |  | PP940118.1 | 99.86 | *E. asburiae* |
| ***Exiguobacterium*** |  |  |  |  |  |  |  |  |  |  |
| T410 | 0.27% (1) | PX096440.1 |  | OTU361 | 0.019% | 100 |  | FJ970034.1 | 99.93 | *E.* *acetylicum* |
| ***Microbacterium*** |  |  |  |  |  |  |  |  |  |  |
| Y005 | 0.54% (2) | PX096441.1 |  | OTU278 | 0.599% | 100 |  | OK035411.1 | 100 | *M. hydrothermale* |
| T064 | 0.54% (2) | PX096442.1 |  | OTU5 | 0.009% | 100 |  | KP980598.1 | 100 | *M. trichothecenolyticum* |
| Y121 | 1.90% (7) | PX096443.1 |  | OTU268 | 1..810% | 100 |  | NR135869.1 | 99.49 | *M. proteolyticum* |
| ***Pantoea*** |  |  |  |  |  |  |  |  |  |  |
| Y013 | 19.78% (73) | PX096444.1 |  | OTU364 | 4.947% | 96.72 |  | MW730583.1 | 100 | *P. agglomerans* |
| Y163 | 1.35% (5) | PX096445.1 |  | OUT303 | 0.048% | 100 |  | PV342444.1 | 100 | *P. dispersa* |
| ***Pseudomonas*** |  |  |  |  |  |  |  |  |  |  |
| T372 | 0.27% (1) | PX096446.1 |  | OTU310 | 4.919% | 98.41 |  | PV612267.1 | 100 | *P. fulva* |
| ***Shigella*** |  |  |  |  |  |  |  |  |  |  |
| Y092 | 0.54% (2) | PX096448.1 |  | OTU349 | 0.091% | 100 |  | ON060619.1 | 99.78 | *S. flexneri* |
| ***Sphingomonas*** |  |  |  |  |  |  |  |  |  |  |
| T016 | 0.27% (1) | PX096449.1 |  | OTU294 | 0.002% | 99.25 |  | OQ652012.1 | 100 | *S. aquatilis* |
| ***Xanthomonas*** |  |  |  |  |  |  |  |  |  |  |
| Y003 | 0.81% (3) | PX096450.1 |  | OTU257 | 11.416% | 100 |  | MN932338.1 | 99.86 | *X. sacchari* |
| ***Total*** | 369 |  |  |  |  |  |  |  |  |  |
